# Factors influencing the preparedness for the implementation of the national health insurance scheme at a selected hospital in Gauteng Province, South Africa

**DOI:** 10.1186/s12913-022-08367-7

**Published:** 2022-08-06

**Authors:** Ntsibeng Valerie Mukwena, Zodwa Margaret Manyisa

**Affiliations:** Department of Health Studies, University of South Africa, Muckleneuk, Pretoria, South Africa

**Keywords:** National health insurance scheme, Implementation, Readiness, Assessment, Challenges

## Abstract

Research studies as well as anecdotal evidence suggest that there are challenges regarding the NHI plan implementation. These include problems such as an increase in illnesses and a shortage of personnel to drive the project in South African public hospitals. This is exacerbated by the existing situation of most government-funded healthcare institutions, which are characterized by bad administration, insufficient budget, inadequate infrastructure, and insufficient drug supply, as highlighted in several studies. The hospital under investigation is one such facility, with a history of patients sleeping on the floor and people being turned away owing to a shortage of experts and an overburdened staff. This situation is concerning, given that the government claims to be providing appropriate funds to the institution. The hospital under research is highly regarded by the surrounding community. However, a visit by the Health MEC in 2014 revealed that the facility had insufficient sanitary standards and a high complaint rate. Based on the foregoing, as well as the difficulties that both employees and patients are confronted with at the  selected hospital, the question that emerges is whether the hospital is fit for the implementation of the NHI.

**Aim:** The aim of this study was to assess the preparation for the launch of the national health insurance scheme at a Johannesburg hospital.

**Setting:** The study was conducted at a hospital situated in eastern suburbs of Johannesburg, Gauteng, South Africa.

**Method:** The study employed a qualitative method with an explorative, descriptive, qualitative study design. The population consisted of selected hospital employees, such as doctors, dispensary officers, hospital managers, human resources workers, facility managers, and administration record officials who were employed at the selected hospital. Purposive sampling was used to select participants.

**Sample size:** Category saturation was used to determine the sample size. The participants for the study were chosen using purposeful sampling, with the researcher aiming for those who were familiar with the NHI scheme at the institution. Semi structured interviews and a focus group discussion were used to gather data. The data from the focus group discussion and semi-structured interviews were analysed using thematic analysis.

**Results:** The investigation found that the hospital was preparing to for the NHI implementation, but that was riddled with lack of resources, poor infrastructure, lack of training, delays in development and poor technological advances.

**Recommendations:** The paper suggests that human resources be increased, infrastructure be upgraded, medicines and equipment be increased, and enough training on NHI implementation be provided.

**Contribution:** The paper adds to the body of knowledge regarding the NHI in South Africa.

## Introduction and Background

Section 27 of the Constitution of the Republic of South Africa, 1996 provides that everyone has a basic right to health care services. However, majority of the South African population does not have access to health care and relies on poorly resourced public hospital to access health care services [1]. This is because they cannot afford private care. These findings are supported by  the South African Human Rights Commission 2019 [1] which found that at least 82 of every 100 South Africans do not belong to a medical insurance hence they solely depend on the public health care. This puts a high demand on the already overburdened public health system as characterized by lack of personnel, overcrowding, long waiting hours unavailability of medication and infrastructure which has an adverse impact on the quality of services [1]

To improve access to health care for the majority of South Africans, the South African government introduced the National Health Insurance (NHI) scheme. The National Health Insurance (NHI) is a health care financing system that is intended to move South Africa, towards universal health coverage and ensuring that the South African population, families, and individuals have access to quality health services care irrespective of their socioeconomic status or standing [2]. The move towards Universal Health Coverage (UHC) through implementation of NHI is derived from the Reconstruction and Development Programme (RDP); the Constitutional mandate based on Sect. 27 of the Constitution and the 1997 White Paper for the Transformation of the Health System.

This move is also underpinned by the vision 2030 of the National Development Plan (NDP), which envisages that by 2030, everyone must have equitable access to an equal standard of health care, regardless of their income. The Vision 2030 of the National Development Plan (NDP) was that establishing common Fund for private and public health providers would enable all South Africans, including immigrants, access to free and equitable health care, regardless of their race or socio-economic status [3, 4]. The NHI was to be implemented in phases, where Phase 1 (2012–2017) consisted of the testing and development of systems and procedures to ensure the health system's efficient operation. Phase 2 (2017–2022) focused on the creation of systems and procedures to guarantee the NHI Fund's efficient operation and administration. Phase 3 (2021–2025) will see the introduction of obligatory NHI prepayment, contracting for authorized private hospital and specialized services, as well as the finalization and implementation of the Medical Schemes Act and the NHI Act. To achieve this goal, a National Health Insurance (NHI) Bill was submitted to Parliament, adopted, and gazetted on the 26th of July 2019. The primary goal of the NHI was to improve user satisfaction, lead to a better quality of life of all citizens and improved health outcomes across all socioeconomic groups. Improved health outcomes of families and individuals will probably contribute towards improved human capital, labour productivity, economic growth, social stability, and social cohesion thus reducing poverty and inequalities inherited in the past [3]. However, this wonderful proposal is fraught with difficulties, as the healthcare system is fragmented and in need of repair as suggested in the Phase 1 of the NHI pilot project evaluation report in south Africa, which revealed that the implementation of the pilot intervention had mixed feelings across the pilot districts. These include the lack of strong political will, inadequate human and financial resources for implementation, poor/ lack of coordination and communication, and a lack of monitoring systems put in place at the time of implementation. Given the existing status of most public hospitals, by the time the NHI program is projected to be fully implemented, the health institutions may still be unprepared.

### Problem statement

According to research, the implementation of the NHI scheme is dependent on a massive reorganization of the healthcare system, which will combine the two-tier public and private healthcare systems into one[5]. With the NHI in place, all health revenue will be deposited into a single fund and nationalised. To achieve this objective, the Department of Health (DoH) chose a few hospitals in provinces across South Africa for its pilot project of the NHI scheme and began implementation in stages in 2012, involving eleven districts in all nine Provinces [6]. The hospital under investigation was among those which were identified for the NHI pilot project. However, according to a report which was compiled in 2019 [7], even though many strategies had been put in place to prepare for the NHI, majority of these hospitals were still unprepared for the implementation of this initiative.

As a case in point, the hospital under investigation is only one among many, where patients have slept on the floor and patients have been turned away owing to a shortage of experts and an overwhelmed staff in previous years [8]. The hospital under investigation is regarded as one of the prestigious hospitals in the local area and in south Africa as a whole because of the government’s claims that it is well resourced. However, this alarming claim were disputed by the health MEC, who during her visit in 2014 confirmed that the facility was under resourced and characterized by poor sanitary conditions and a high proportion of complaints [9].

Weighing in on all of this is a legitimate concern about whether the selected hospital can successfully transition to an NHI program, given its existing financial and operational challenges. Despite these difficulties, very little research has been done to examine the preparedness of hospitals for the implementation of the NHI. From the problem statement. the following objectives emerged:

- To explore and describe the factors influencing the preparedness for the implementation of the National Health insurance at a selected hospital in Gauteng Province, South Africa.
- To propose recommendations that will facilitate effective implementation of the NHI scheme at the selected hospital in Gauteng Province, South Africa.

## Materials and Methods

An exploratory, descriptive, qualitative study design was used to explore and describe the factors influencing the preparedness for the implementation of the NHI at a hospital in Gauteng province, South Africa. The researchers opted for an exploratory, descriptive, qualitative design for this research. A qualitative design involves studying things in their natural settings in an attempt to interpret them in terms of the meanings people bring to such settings [10]. Qualitative designs are valued means that are used to explore the subjective experiences of participants about a certain phenomenon or a topic with limited coverage within the literature thus allowing the participants of the study to contribute to the development of new knowledge in that [10, 11]. Furthermore, literature suggests that qualitative research is designed to illuminate how a phenomenon is manifested and is especially useful in uncovering the full nature of a little-understood phenomenon. In this research, the qualitative methos were deemed appropriate for exploring and describing the experiences of departmental managers regarding the factors influencing the preparedness of the selected hospital for the implementation of the NHI as they are concerned with answering questions such why, what and how in healthcare setting.

The probing nature of the research provided the researchers with a rich insight of the strengths and challenges that the employees must deal with in their endeavours to achieve smooth implementation of the NHI in South Africa.

### Study setting

The study was conducted at a selected public hospital in the east of Johannesburg, Gauteng Province, in South Africa. This hospital is one of the hospitals that were selected for phase 1 implementation of the National Health Insurance scheme in South Africa. The hospital was chosen because it is a regional hospital, and services a greater population of Johannesburg, which has just over six million occupants. The authors aimed to capture the essence of the NHI issues based on the client base of the hospital, and its status as a regional hospital. The hospital was chosen because it has a dedicated quality assurance department, which deals with the ideal hospital framework, which is the heart of the National Health Insurance framework.

### Study population and sampling procedure

The target population for this research comprised 15 hospital sectional heads or managers, namely; nursing, medical services, quality section of the hospital, dispensary, hospital managers, human resources section, laboratory and administration records officials who were involved in the implementation of the National Health Insurance (NHI) scheme at the selected hospital. There is a lot of value in looking at department leaders, since they have a better understanding of their departments than their subordinates].

Purposive sampling was used to select participants for this research. A purposive sampling technique is defined as the deliberate choice of participants due to the qualities they possess [12].

For this research, the researchers deliberately selected individuals that were considered to be proficient and well-informed with the preparation processes for the implementation of the NHI scheme at the selected hospital. Individuals who were not involved in the processes of implementation of the NHI scheme as well as those who were on leave for more than three months before data collection were excluded from the research.

### Sample size

The sample was made up of eight hospital departmental heads from the human resources, quality assurance, administrative, finance, nursing and dispensary departments.

The sample size was determined by the availability of officials in each department who were directly involved in the implementation of the NHI, in their capacities as the departmental heads. After applying the inclusion and exclusion criteria and factoring in availability of participants for the study, the sample consistently became narrower. Of the accessible population of heads of department, only eight agreed to participate in the study. The size of the sample was considered to be adequate enough to enable the extraction of thick, rich data as suggested in literature [13] mainly because it was based on the concept of information, which suggests that, the more relevant information power the sample holds, the smaller the sample size is required for the research and vice versa [14]

Focus group discussions and semi-structured interviews were used to collect data for this research. All the 8 participants that took part in the semi structured interviews, also took part in the focus group discussions. The participants were split into two groups based on their availability. Focus group discussions involve collection of persons who have a common interest or background who meet to discuss that subject [15]. In this research, which comprised a smaller sample size, focus groups proved to be a rich source of information and  was easier to manage as suggested in several research studies. 

A semi-structured interview is a discussion between the researcher and the participants that is led by a flexible interview technique and augmented by follow-up questions, probes, and remarks [16–18]. In this research, an interview guide was used to obtain data from the participants. The interview guide was developed based on the objectives as well as the relevant literature review. The interview guide was piloted through interviews with a small group of managers with similar characteristics as those of the participants, but who did not form part of the research and was modified until the researchers were confident that it addressed the objectives of the research. Data collection took place during the Covid 19 pandemic between April and June 2021, therefore, to adhere with the National department of health Covid19 regulations in South Africa, virtual sessions on Microsoft Teams were arranged with the participants for both the focus groups and the semi-structured interviews. The purpose of the research was explained and informed consent was sought from all participants before data collection commenced. In addition to that, the principles of autonomy, respect, confidentiality, beneficence and non-maleficence were observed throughout the research process. During the interviews. the interviewer asked a broad question, to begin each discussion, filtering down to probing questions based on each group interviewee’s experiences. To ensure quality and validity of the data, an audio recorder was used to capture the data with the permission of the interviewees. Field notes were also taken during the process of interviewing to enhance the accuracy of the data [17, 18]

### Data analysis

The results of the focus group discussions and semi-structured interviews were analysed using thematic coding [19]. The emerging themes were identified, and notes made on the transcripts using the following steps; 1) generating initial codes, 2) searching for themes 3) reviewing themes 4) defining and naming themes s and 5): producing the report.

After the analysis, the data was transcribed using procedures that subscribed to the UNISA code of conduct as well as ethics. The transcription was made using the Atlas ti UNISA license. The MP3 recordings and transcribed data were kept securely locked up in a place and were  only accessible to the researchers, while the soft copies were stored in a password protected computer. The relevant knowledge obtained was organised and interpreted in the results section of the study.

### Trustworthiness

Trustworthiness or rigor of a study refers to the degree of confidence in data, interpretation, and methods used to ensure the quality of a study [20]. The researchers adhered to the five criteria to develop trustworthiness in qualitative research namely; credibility, dependability, confirmability, transferability, and authenticity. These criteria are briefly described in the next section as follows: [20].

Credibility is defined [20–22] as an accurate portrayal of participants. To ensure credibility of the research findings, the researchers adhered to the prescribed design and methods of qualitative research as suggested in literature [20, 21]. In addition to that, the researchers provided a summary of the themes that emerged from the interviews and requested feedback from the participants to validate the researchers’ conclusions. Dependability refers to the constancy of the data over similar conditions [21]. Dependability was ensured by providing a clear description of the designs and methods to allow other researchers to replicate or reproduce the study [22].

Confirmability refers to the researcher’s ability to demonstrate that the data represent the participants’ responses and not the researcher’s viewpoints [22]. To adhere to the criterion of confirmability, the researchers provided a clear description of how conclusions and interpretations were established, and by providing enough evidence that that the findings were derived directly from the data as presented by the participants.

Transferability refers to the capacity of a study findings to be applied in another context [22, 23]. To adhere to this criterion, the researcher provided detailed information regarding the research participants and the context to allow other researchers to assess whether the findings are transferable to other settings as described in literature [22, 23]

## Results

Six major themes emerged during the data analysis process as follows resources, infrastructure, training, technology, development, and management. The themes on factors influencing the preparedness of the selected hospital for the implementation of the national health insurance scheme are displayed in Table1.

**Table 1:** Themes on factors influencing the preparedness of the selected hospital for the implementation of the national health insurance scheme at a hospital in Gauteng Province, south Africa

Theme	Subtheme
1. Lack of resources	1.1 Shortage of staff and specialists
	1.2 Lack of funding
	1.3 Insufficient beds
	1.4 Lack of equipment
2. Poor infrastructure not meeting the needs of patients	2.1 Old and inadequate facilities
3. Lack of participation in training	3.1 Lack of definitive training for the NHI
	3.2 Fewer specialists being trained
4. Poor technological advances	4.1 Paper-based record management system
	4.2 Outdated laboratory equipment
	4.3 Monitoring systems
5. Stalled development	5.1 Ideal hospital Framework
	5.2 Expansion stalled
	5.3 Systemic pressure
6. Management failures	6.1 Procurement processes
	6.2 Job dissatisfaction

### Lack of resources

Participants discussed how the hospital was affected by lack of resources. They cited lack of human resources as a major challenge at the hospital under investigation. The participants expressed their frustrations about the lack of specialists. They attributed the lack of human resources to poor salaries. They explained that medical specialists and hospital management personnel were underpaid and as a result, were flocking to the private sector in large numbers. One participant said:

"…the department does not pay very well, so specialists are leaving for greener pastures, which is a problem because we need them to prepare our hospital for the impending NHI”

A common theme that emerged during the discussion was the Department of Health's lack of funding. Participants explained that the hospital under investigation is heavily reliant on government and parastatal funding. A participant who is well-versed with the hospital's administrative and managerial processes mentioned that there were many opportunities for improvement at the hospital, but the institute was lagging in addressing them due to lack of funding. The participant said:

*"The hospital does not have the money to address the areas of improvement as they are identified*. *If the hospital were proactive in addressing its issues, rather than reactive, we would have achieved the ideal hospital framework by now,”*

The above discussion suggests that the participants recognise a pattern of raising concerns at the hospital, that are never addressed.

The number of beds in the wards was a recurring theme in the responses by the participants. The beds are of mediocre quality, and they are rarely serviced. Given the number of patients admitted to hospital wards daily, the number of beds available is insufficient. Some participants stated that it was impossible to ensure patient safety when patients were crammed together in wards, on floors, on stretchers, and, if they were lucky to find beds.

The participants raised a critical issue regarding the equipment that is used in the hospital. They all emphasised that the hospital did not have the equipment to cater for every condition that patients are bound to bring for medical attention. It is worth noting that the selected hospital is a regional hospital that serves most of the city's central townships. The fact that the hospital must borrow equipment that is important to health-care delivery, as described by the participants, is concerning, as it suggests that the hospital may be deficient in basic technology that any health-care provider is supposed to have.

### Poor infrastructure

Poor and dilapidated infrastructure was a strong sentiment that was echoed by most of the participants when they were deliberating on the readiness of the hospital for the implementation of the NHI. From the discussion related to facilities participants indicated that some facilities at the hospital are outdated and no longer meet the demands of the patients being served. The participants indicated that, even though there is a specific portfolio in this respect, it receives limited aid from the authorities. They mentioned that some facilities such as waiting areas, wards, specialists' rooms, theatres, and surgical rooms, require urgent modifications to better meet the requirements of current patients. Most participants spoke passionately about the issue of facilities, highlighting a problem that has been mentioned but not solved on several occasions. The participants were concerned that the space issue might reoccur after the NHI was completely implemented as more patients would be serviced at the facility.

The participants attributed the poor infrastructure to the fact that the selected hospital was built before South Africa attained its freedom hence was no longer satisfying the needs of the patients who are being served. While the hospital was originally an impressive institute, it has not kept up with the modern developments that other world-class institutions have undergone. They explained that even though there is a dedicated portfolio in this regard, it receives minimal assistance from the authorities.

The participants said:

“*The plumbing at the hospital is old and needs to be updated. How can we transition to a universal healthcare system, when we do not have basics, like a modernised plumbing system?”*

Another participant went on to describe the current state of the facilities at the hospital as follows:

“*There is not enough space for the number of people that get admitted to the wards. Some people must sleep on the floor, on stretchers, and outside, all because of space constraints. Surely this shows that the NHI is a commitment that the institute is not ready for, not by a long shot.”*

*“The hospital does not have functional emergency services. There has been only one ambulance serving the hospital over a 30km radius*.”

The above quotes indicate that the participants were all convinced that for the hospital to become NHI-ready, the issue of space needed to be addressed. Most of the participants addressed the issue of facilities with passion, pointing to a problem that has been repeatedly raised but not addressed. The participants were anxious that the space issue would recur when the NHI is fully rolled out, as more patients would be served at the institution.

### Lack of participation in training

Participants mentioned that there were training gaps in the hospital on the NHI, training required for the hospital employees, and educating medical expert staff to provide the best treatment to patients. Participants expressed a variety of opinions on the training of relevant parties at public health institutions in preparation for the NHI's implementation. From this theme, the subthemes namely; lack of participation in training and fewer specialists being trained emerged: The subthemes are described in the next section.

The participants appeared to concur that the hospital staff had received no definite training about the NHI. They explained that, while the staff was aware of the DoH's external push to prepare the institution for the NHI rollout, they had not yet received definitive training in their various sections for the actionable items they needed to close out to prepare the hospital for the universal healthcare scheme.

Participants stressed that the hospital needs to find ways to train additional experts because, as previously stated, professionals were departing the public health sector for better pastures at a higher pace than the new employees. This demonstrates that the participants are aware of the organisational inadequacies, particularly, specialized personnel shortages, that may impede progress toward the NHI.

- The participants indicated that because the departments work in silos, there has been a breakdown in communication that would be beneficial in ensuring that the scheme's preparations are done uniformly across the hospital campus. They also mentioned that they felt they were excluded from key programs that have an overall impact on the hospital's operation. One of the participants said:

*"We are not consulted to give our input for issues that we still have to take part in. I am sure that a number of us have a lot of ideas that if taken into consideration, would better the status of this institute”*

### Poor technological advances

The participants felt strongly that the hospital lacked modern technology and other improvements that are required for healthcare to provide the greatest quality of treatment. More particularly, the participants were worried about the hospital's record management system, laboratory equipment, and monitoring and evaluation, which developed as subthemes under this issue. The subthemes are discussed in the next paragraphs.

Several interviewees indicated that the hospital still employs a paper-based record management system. This generates multiple hitches from the patients' admission through discharge as records must be kept and used at all phases of the procedure. During the process, records might get dirty, ripped, lost, or misplaced. Most participants felt that the paper-based record system was an issue that may potentially delay the hospital's preparation for the NHI. The (NHI) is a universal healthcare program that requires public and private healthcare providers to be connected so that patients may be readily sent to different experts within the same network. Participants reported that there were sometimes hiccups in the admissions process owing to paper-based records.

One participant said;

“*Sometimes patients wait for long periods, to access healthcare, because of menial issues like lack of stationery for the admission process. If not that, there is sometimes miscommunication between the healthcare professionals based on what is documented on the paper records, which results in errors that could have been avoidable had the system been electronic*.”

“*There is a clear effort from the Department of Health to improve the information management system, however, this is an initiative that is lagging behind.”*

“ I do think the there's been a clear effort from the department to try and transition the sector, the *public sector as a whole from being more paper based to electronic based. I mean ehhhh…. I mean pharmacies can have the automated press button retrieve medication. So, when I think it’s a very broad to say, but what I would say I do believe at this current moment, yes, we are very, paper-based just looking at basic patient records.”*

*“Like I said, a clear move towards a more electronic management of data and information so much so that I think in the past two months they have been workshops that have been helped virtually because the department is trying to choose an electronic system of record keeping. So, there's already other systems in place. For example, in terms of x-rays, there's something we call the tech system where if there's an x-ray done, they can be viewed in another area that's already electronic. So, they have been advances already. But I think the, the, the biggest move is going to relate around the record keeping and, and data management. So, it's not where it should be yet, but the initiatives are there to implement it. So, will it be there by 2025? I think definitely.”*

During the conversation, one participant expressed a special concern about the old and non-functional laboratory equipment, stating that it needed to be either reconditioned or replaced, depending on its condition. The participant said:

*‘The laboratory is a cause for concern. Some of its equipment no longer works, and some have gone redundant. To update the laboratory requires a massive cash injection, and this is something that the Department of Health does not have now. Remember, the laboratory is an essential part of the hospital, as we do most of our tests there for proper diagnosis. Referring patients to another service provider for tests is a time-consuming activity, which is a luxury that most patients do not have. If we are to provide services under the NHI scheme, then the laboratory would need to be attended to.’*

It is worth noting that this participant chose an area that is deficient to explain their perception of the hospital's readiness for NHI adoption.

Poor monitoring systems was also cited by the majority of participants. According to participants, the monitoring systems in the selected hospital have not been as successful as they could have been. The poor monitoring systems were attributed to factors such as shortage of resources as well as other complicating variables such as the coronavirus pandemic that the hospital was experiencing at the time of the research, hence, the hospital has been functioning in crisis mode. Participants admitted that they had not manageg to do monitoring and evaluation as they had been focusing less on the systems and more on handling the hospital's issues. They indicated how the COVID19 pandemic has impacted the already overburdened staff from doing the preparations and from conducting monitoring and evaluation of all the hospital processes as per the guidelines.

The above-mentioned claims are evident in the following quotes by the participants:

“The IDEAL hospital framework assessment has not been performed since 2019 due to the COVID pandemic, but during the last assessment, the hospital scored less than 50%.’’.

‘’The thing with COVID is that it tests so many different facets of the health system. It's not pure a silo effect where we say, how has it affected this hospital.”

*“When you're in a pandemic, remember it's all hands-on board. So, all resources in terms of time, in terms of warm bodies slash HR in terms of equipment all of that needs to be challenged and that's just the nature of a pending. So same as, as wartime... all that needs to be channeled towards fighting, whatever the pandemic may be. So going back to the ideal hospital assessment and how the....., that could directly be affected as the assessment in itself remember needs warm bodies. I mean, it is staff members within the institution who are briefed to say, this is the 20-page assessment for this next week, we're going to go to this area. You will go to this area and check this and check if this is present, check, if this is not there.”*

“*It’s a very extensive assessment that looks at every facet of the running of an institution. If I were to share the book with you, which I hope I can, after this interview, it's a textbook that says infrastructure. These are the things in terms of infrastructure. These are the markings that should be paid for a safe hospital. You know, these are this is the type of equipment that needs to be there. These are the processes or systems that need to be placed where they be checking resuscitation trollies every morning, whether it be making sure that a certain equipment is not placed there, but it's placed there. It's very detailed. So that's why you find that the assessments in themselves can take up to a whole week to assess the entire institution.”*

### Stalled development

Most participants indicted their dissatisfaction regarding the lack of developments at the hospital. The participants debated the notion that there had been tiny progress at the hospital during the previous 15 years. They mentioned that despite the changes in the patient demographic and economic position, there is no visible lack of expansion of the hospital to accommodate the needs of the patients. The lack of expansion of the hospital was attributed to a number of factors, including budget cuts and corruption. The statement regarding the lack of substantial modifications being made to the hospital was echoed by a participant who has worked at the institute for over two decades, who stated that there have been no changes or any improvement made to the hospital infrastructure, equipment, or human resource structure throughout his tenure. 

A participant who had been with the institute for more than 20 years described the pattern of events at the hospital. She said:

*“There are constant promises from the government about bettering the hospital, in terms of staff complement, equipment and general support. Every year, the same song is sung, but nothing ever really changes*”.

The findings presented in this paper revealed that the hospital was not meeting the ideal hospital framework criteria. The participants described the ideal hospital quality improvement plan is a live document, that is being manned by four quality assurance employees, but the progress for its practical implementation is insignificant.

The National Department of Health devised a concept known as the ideal clinic, which comprised a set of ideals that applied to healthcare practitioners such as adequate resource namely, finances, human resources, good infrastructure as well as policies. It explains the fundamentals of what makes a world-class healthcare practitioner. The findings of the present research discovered that the ideal hospital structure is comprehensive in the eyes of the participants. Another participant suggested that following the conditions of the framework stated previously, let alone achieving its aim, was challenging. The ideal hospital framework’s goal is to prepare hospitals for the adoption of the NHI system. However, the participants felt that although they were putting a lot of effort to get the hospitals ready for the implementation of the NHI, more work still needed to be done as they were hindered by a number of systemic challenges that they needed to overcome which could take longer than anticipated. They felt they needed more time to prepare the hospital as per the guidelines for the implementation of the NHI scheme.

One participant said.

“*The route to NHI implementation, it’s not going to be a case where you know, today it’s a public sector versus private sector and then all of a sudden, tomorrow we are completely merged. It’s going to be a journey where we then try and sort of equalize ourselves in terms of resources, where you’re trying to merge two very different systems together. So, it’s not an overnight or even a one-year thing. So, what public institutions are doing or involved with is that there is something called the ideal hospital framework that each hospital tries to realize.”*

*“From what I have heard, what I know is that, unfortunately, we were not able to realize ideal hospital status at the last assessment, which was last year late 2020, around November. So, eh, basically what that says is that there were certain vital areas that needed to be corrected before we can be considered to have achieved ideal status. And the thing with ideal status you know, these separate categories of I do this platinum, this gold, but we were not able to achieve status at all at our last assessment*.*”*

Another one said:

“Because remember the health system is interlinked, you need a functional emergency services. For example, you need a functional primary *health care network to feed into the institution. So, it's not just to say, how has it affected the hospital in isolation, It is to also say, we must consider how the effect of COVID … at the facets of the health system have an impacted on our hospital.”*

### Management failures

Some participants complained that the procurement processes for services and goods were excessively time-consuming as they were hindered by red tapes. They attributed these procurement challenges to management failures. They also discussed supply chain limitations, which they believed were responsible for the hospital having a temporary scarcity of vital medication. This was evident in the following quote: *“It is unfortunate that most shortages of medication are systemic in nature, because the system is designed in such a way that it impedes its own progress.”*

Another participant said:

“The number of hoops that one has to jump just to be able to get a service provider into the system. Besides, the red tape that is associated with payments for services rendered would shock you”.

### Increased workloads causing job dissatisfaction

Participants voiced their concerns about heavy workloads and how it affects their views toward the NHI. They said that their minds had not yet been conditioned to manage the National Insurance Scheme. The participants also stated that they believed that increased remuneration for their work would encourage them. They explained that they were not motivated to be fully involved in the NHI preparation process as they were already struggling with their workloads and were unhappy with that. They stated that the current system can not cope with the patients’ workload as exacerbated by the Covid 19 pandemic and staff shortages which has overburdened the patient transport systems. They mentioned that the present systemic situation has created an unsafe environment as those who are critically ill have to wait longer than necessary and end up losing their lives. One participant said:

*“I am unhappy with having extra work, as I already have my hands full with what is happening in my department. There is always something happening at each point, I am not sure that adding extra work is a particularly clever idea now.”*

*Another one said:*

“Patients are having to wait longer for service, leading to early demise because there is no manpower.”

Another one echoed the following statement:

“*So obviously patients are waiting longer. Patient has to wait longer because by the time they arrive here, maybe they're critically ill. And let's say within an hour, two they've demised. So that's a systemic pressure that has resulted. So the death will be at this hospital, but it's a systemic pressure sort of resulted in an unsafe in, in us almost being an unsafe institution.”*

## Discussion

This section focuses on the discussion of the findings against literature review.

### Resources

Lack of resources emerged as a major issue at the selected hospital as most participants reported that the hospital lacked both material and human resources. The lack of resources was attributed to the lack of a budget, which is essential to alleviate the lack of equipment and supplies, as well as the shortage of employees, which resulted in lengthier waiting times. The findings presented in this paper revealed that the situation is so grave that patients are forced to sleep on the floor. This has had an impact on the hospital's preparations for the NHI's implementation. A number of studies have revealed that the lack of resources is a reoccurring issue across the country since most public hospitals are in the same boat [24, 25].

Budgetary constraints were identified as a serious challenge as participants mentioned that there is no sufficient budget to acquire essential modern equipment to make patients’ critical diagnosis at the selected hospital. The lack of equipment was attributed to inadequate financing from the central office (DoH), which made it impossible to modernize equipment, acquire consumables, and improve the institute's healthcare quality. A study on public health concerns in the Free State [26] found that financing from the DoH was insufficient and usually project specific. The study also discovered that the financing was inequitable since it did not consider the hospital's requirements and distributed funds evenly throughout the public hospital system. This might be one of the reasons why the selected hospital is unable to handle its cash effectively.

The findings of this research also suggest that the facilities in the selected hospital under investigation are outdated as is evident by the participants’ remarks that the institute had not grown significantly in the recent two decades. Given the changing demographics of the patients served by the institute, the hospital requires improved facilities, if not an extension to the current structures. Similar findings were reported in a research on working conditions in public hospitals in Mpumalanga Province which described how most facilities in South Africa were old and decrepit, producing hazardous and unclean circumstances for the already suffering patients [24].

The NHI system intends to cover all South Africans, including long-term foreign immigrants. This indicates that when the NHI is implemented, hospitals will cater for a greater number of patients. However, participants expressed their concerns that the hospital does not have the infrastructure to absorb an additional number of patients because it is currently congested with patients. These claims are supported by a study on clinical teams' experiences of overcrowding in public emergency centres in Cape Town, which reported overcrowding as a major problem in public health institutions [25]. The findings presented in this present paper are further corroborated by a study which reported that public-hospitals, which cater for the majority of South Africans are faced with inadequate and poor infrastructure as well as human-resourcing ratios in relation to the increased number of patients that they have to care for. [29. The problem of inadequate and poor infrastructure at the selected hospital is cause for concern and needs to be prioritised if the hospital is to be ready to implement the NHI scheme.

Lack of training was identified as another factor that influenced the implementation of the NHI scheme at the selected hospital as most of the sectional heads revealed that that personnel at their institution had not been educated on how to administer the National Health Insurance (NHI). For the NHI initiative, a specific department was trained to evaluate the best hospital structure, however, they felt that because the system is being introduced across the hospital, each department must be educated on their respective responsibilities. Lack of training and the departments' functioning in isolation have been cited as two of the main difficulties. The findings presented in this paper are consisted with another study which revealed that heads of various departments at hospitals in south Africa function in silos and that these has resulted in a lack of coordination and trust across the departments in most South African public hospitals [27].

Human resources training is critical for the NHI's implementation to be a success as suggested by a study which focused on South Africa's alignment of strategic human resources towards the NHI, whose findings revealed that more resources for subject-matter training were required [28]. Participants lamented about the scarcity of skilled personnel across the board. The findings reported in this paper regarding scarcity of skilled personal across the board are consistent with another study on the challenges encountered by healthcare personnel in Limpopo Province, South Africa, which revealed a dearth of highly qualified staff can be attributed to a steady decline in the number of medical students enrolled in residency programs [29].

The findings reported in the present research paper revealed that technology was a problem at the selected hospital as the participants strongly felt that the hospital was lacking in terms of advancements that are necessary for healthcare to provide the highest quality of healthcare. The findings are in line with a number of other studies which reported that participants were mainly concerned about the poor record management, equipment as well as monitoring systems in public hospitals in South Africa [30].

Another study reported similar findings which suggests that patients’ health records in public hospitals are not integrated into a single system and as a result, patients are expected to open a new file every time they visit another hospital. The lack of integration of health records impacts negatively on the quality of patient care. This also compromises the quality of the medical services as the doctors  have to diagnose patients without the medical history [31].

Poor technological development is another challenge that emanated from the findings of the study. According to the findings of the investigation, the equipment at the hospital was obsolete and antiquated as the equipment in the departments has not kept up with medical advancements and that only a few of the hospital wards have modernized equipment. For example, the laboratory equipment at the hospital has not been updated or calibrated in a long time as a result, staff were concerned that the device may malfunction, resulting in misdiagnosis and legal actions from patients. These findings are consistent with the findings of a research conducted on the experiences of nurses regarding a significant lack of medical equipment in a rural district hospital in South Africa, which reported that the equipment  at the hospital were of poor quality and were not well maintained [30]. The previously stated study [30] highlighted a similar occurrence that lack of basic medical equipment jeopardizes patients' lives and leads to poor patient diagnosis. The lack of modernized equipment at the selected hospital is cause for concern and needs to be addressed urgently as mentioned by the participants that if the hospital under investigation continues to lack equipment, it will not achieve its envisaged goal of executing the NHI.

The findings presented in this paper suggests that the hospital does not meet the criteria of an ideal clinic/hospital framework An ideal hospital framework is defined as “ a hospital which is in a good physical condition with infrastructure and spaces, essential equipment, and information and communication tools, adequate staff, adequate medicines and supplies, good administrative processes, and adequate bulk supplies and uses applicable clinical policies, protocols and guidelines, as well as partner and stakeholder support, to ensure the provision of qualityhealth care [31]. From the findings presented in this paper, it is evident that the hospital under investigation does not have most of the essential resources as outlined in the definition of the concept of an ideal hospital framework.

The paper reveals that some characteristics of the hospital's administration had the impact of undermining the hospital's preparations for NHI adoption. Increased workloads and poor pay leading to job dissatisfaction was widespread among the primary concerns identified by the participants. The problem has resulted in a workforce that is uninspired to do their jobs, much alone undertake a substantial shift such as the implementation of the NHI. The employees are unwilling to put in extra work until their pay is increased. The findings of the present research are consisted with  a study on the degree of job satisfaction and the impact of discontent on workers retention among nurses in the North-West Province [32]

The investigation revealed that the hospital's departments were not working together. This had an impact on the institution's planning and preparedness. Fragmented efforts in terms of the steps required to implement the plan have hampered the institution's overall preparation for the NHI. These conclusions are supported by another author [33], whose analysis of the historical foundations of the present public health concerns in South Africa, highlighted a lack of stewardship in hospital departments as well as a lack of coherence.

In addition to the above challenges, procurement processes at the hospital appeared to be time-consuming and regulated by a plethora of unnecessary policies. Because of this, the researchers discovered that it was difficult to procure hospital supplies and equipment. The lengthy procurement processes were attributed to management failures which according to the participants, have hampered the preparations for the NHI's implementation, as supplies are frequently delayed and of inferior quality. These findings are in line with another researcher’s findings [34] who in their study, found that there were drug shortages which were caused by procurement processes in the public health sector in South Africa. The study [34] also revealed how procurement processes may have a detrimental influence on hospital operations if they are clogged with policies.

The challenges regarding the preparations for the implementation of the NHI identified in the present paper are similar to the findings of other studies that were conducted on a similar topic. For example, studies which explored the views of public service managers on the implementation of National Health Insurance in primary health care facilities in Johannesburg found that South Africa has failed in several previous attempts to implement structural and financial health reforms [35, 36]

### Strengths and limitations of the study

- Most of the views of the participants were of a systemic character and could thus be applied to other public hospitals in the same region. The findings are solely relevant to the institution under investigation and cannot be generalized to other institutions
- The researcher could not obtain a larger sample size as only eight people who were directly involved with the implementation of the NHI scheme participated in the research. The authors believe had the sample been bigger, the outcomes would have been different
- The responses provided by the participants in the focus group discussion may have been affected by group thinking and mob psychology rather than based on true individual opinions.

## Conclusion

The study revealed that the selected hospital has made some significant progress in the preparations for the implementation of the National Health Insurance (NHI) scheme. However, even though the hospital has made some progress, the evidence provided in this paper indicates that to make sure that the hospital is ready for the insurance scheme, the implementation team heavily relied on the ideal hospital framework as a gap analysis tool. The National Health Insurance Policy and the Bill have a lot of gaps in terms of how to effectively prepare and implement the scheme. In the process of preparing for the implementation of the NHI scheme, it was revealed that the hospital lacked a site-specific plan because the institution failure to address issues like resource allocation, training, infrastructure, and development, the spread of NHI preparatory requirements was was found to be a confounding element.

This paper suggests some factors that influence implementation of the NHI scheme include lack of resources, such as hospital equipment, human resources/labour, and finances from the central office (DoH). The hospital's inability to offer excellent service as characterised by long waiting hours, suggests that the hospital may be unable to cope if the NHI is implemented. The findings reported in this paper are corroborated by the most recent ideal hospital framework review that took place at the hospital in 2019, which revealed that the hospital's conditions were inadequate and thus unprepared to execute the NHI scheme [36]. In addition to the abovementioned challenges, the continuous use of paper-based systems at the hospital shows that the hospital is not yet ready to implement a high-level medical system like the NHI fund, which would be better served by a fully computerized and automated information system instead. The findings of this paper suggest a lot of work has been done by the hospital, but it still has a long way to go before it is ready for the implementation of NHI. 

## Data Availability

The authors confirm that the data sets generated and analysed during this study are not publicly available since the manuscript stems from a dissertation submitted by NV Mukwena in partial fulfilment of the requirements for the degree of Master of Public Health at the University of South Africa; Supervisor: Dr ZM Manyisa.
